# An Investigation into Spike-Based Neuromorphic Approaches for Artificial Olfactory Systems

**DOI:** 10.3390/s17112591

**Published:** 2017-11-10

**Authors:** Anup Vanarse, Adam Osseiran, Alexander Rassau

**Affiliations:** School of Engineering, Edith Cowan University, 6027 Perth, Australia; a.osseiran@ecu.edu.au (A.O.); a.rassau@ecu.edu.au (A.R.)

**Keywords:** neuromorphic olfaction, electronic nose, biomimetic sensors

## Abstract

The implementation of neuromorphic methods has delivered promising results for vision and auditory sensors. These methods focus on mimicking the neuro-biological architecture to generate and process spike-based information with minimal power consumption. With increasing interest in developing low-power and robust chemical sensors, the application of neuromorphic engineering concepts for electronic noses has provided an impetus for research focusing on improving these instruments. While conventional e-noses apply computationally expensive and power-consuming data-processing strategies, neuromorphic olfactory sensors implement the biological olfaction principles found in humans and insects to simplify the handling of multivariate sensory data by generating and processing spike-based information. Over the last decade, research on neuromorphic olfaction has established the capability of these sensors to tackle problems that plague the current e-nose implementations such as drift, response time, portability, power consumption and size. This article brings together the key contributions in neuromorphic olfaction and identifies future research directions to develop near-real-time olfactory sensors that can be implemented for a range of applications such as biosecurity and environmental monitoring. Furthermore, we aim to expose the computational parallels between neuromorphic olfaction and gustation for future research focusing on the correlation of these senses.

## 1. Introduction

The need to detect the presence of hazardous volatile organic compounds (VOCs) first arose during the Industrial Revolution and sparked research in gas sensing technology where, initially, the gas sensors were mainly implemented as mechanical devices [1,2]. Over the last few decades, the market for gas sensors has been steadily increasing with particular interest in developing inexpensive, small, real-time and accurate gas sensing systems [3,4]. Recently, the application scope of gas sensing devices has been extended to other fields such as food safety, bio-security, healthcare and air quality monitoring. Advances in electronics and computing technologies, along with the increasing scope of application, led to the development of more sophisticated electronic gas sensors [5].

The introduction of bio-inspired olfaction methods by Persaud and Dodd [6] exposed an entirely new pathway for the development of electronic nose technology. Gardner and Bartlett defined the electronic nose system in [1] and identified the sensing front-end and the pattern recognition-based processing unit as key components for such devices. Sensing front-ends such as chemiresistive sensors are largely utilized in common e-nose systems. The transduction principle of these chemiresistive sensors is based on the change in resistance due to chemical interaction with different odours [7]. The chemically interactive material employed in such systems is mainly metal-oxides (MOX) or conducting polymers (CP) [8]. Metal-oxide sensor arrays are among the most widely used sensing front-ends, mainly because of their short response time, high sensitivity and low-cost [8]. One of the major drawbacks of these sensing arrays, however, is their excessive power consumption due to the high operating temperatures required. Moreover, the output of these sensors is susceptible to environmental changes [9]. Conducting polymer sensors are popular for low-power implementations that require high discrimination capabilities; however, their application is limited by the drift caused by changes in humidity [5]. Recent progress in electronic sensing technology has resulted in the development of novel sensing methods based on micro-electro-mechanical systems (MEMS), carbon nanotubes (CNT) and piezoelectric sensors [7,10].

The information generated by the sensor array could result in a considerable data overhead. As a potential solution, pre-processing techniques such as dimensionality reduction and feature extraction can be implemented in conjunction with conventional pattern recognition methods for the classification and identification of odours [11,12]. These data-intensive processes require substantial computational power and tend to incur considerable latency, which hinders the real-time operation of the e-nose systems. Recently, spike-based neuromorphic approaches have led to the development of novel processing solutions for sensor systems, especially for vision and auditory applications [13,14]. Current artificial olfaction systems emulate the neurobiological architecture of the olfactory pathway. The introduction of neuromorphic approaches enables the representation of sensory data into sparse spikes that could encode critical information for classification and identification of odours [15,16]. Low-power spike-based processing and the ability to embed learning algorithms underpin the application of neuromorphic systems for the development of robust real-time e-noses. In this paper, we review and analyse major contributions in both conventional and neuromorphic e-noses to identify current trends and the scope for future developments.

## 2. Conventional E-Nose Systems

In 1982, Persaud and Dodd [6], proposed a model of an electronic nose that established a generic architecture for bio-inspired machine olfaction systems. This study emphasized mimicking the three-level processing of the biological olfactory pathway, which consists of odour receptor neurons (primary neurons), glomerulus (secondary neurons) and the olfactory cortex. The functions of these levels were emulated using sensing elements, an amplifier that measures and evaluates sensor response based on a defined algorithm and a window comparator for classification of the odour. In order to replicate the high selectivity of mammalian olfaction, multiple semiconductor gas sensors were organized in an array to exploit the overlapping sensitivity towards different Volatile Organic Compounds (VOCs). This method facilitated effective discrimination between complex odours.

**Figure 1:**
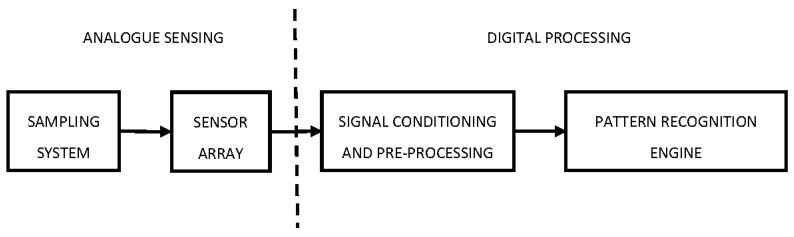
Key components of an e-nose system.

The term ‘electronic nose’ coined in [6] was formally defined by Gardner and Bartlett in [1]. This definition identified the key building blocks in an artificial olfaction system that can discriminate between a number of simple and complex odours. The authors describe an electronic nose as a device comprising of an analogue sensing unit and a digital processing unit [17]. An odour delivery system and a sensor array formed the analogue sensing unit, whereas the digital processing unit included an interface for sensing elements that performed analogue-to-digital conversion (ADC), array processing for the normalization of sensor responses and a memory-based pattern recognition engine that used previously stored odour ‘fingerprints’ for classification and output prediction (Figure 1).

In later work, Gardner et al. proposed integrating CMOS gas sensor arrays and processing units on a single chip to develop intelligent olfactory systems such as the nose-on-a-chip [3]. This provided a practical design for an integrated intelligent sensor that could reduce power consumption and provide better control over undesirable variations when implemented in a real-world application. This study particularly highlighted the quintessential properties and functionalities of the integrated smart sensors for electronic noses and addressed the challenges of applying them in a real-world environment. Subsequent research on artificial olfactory systems was largely inspired by the analysis and architectures outlined in these early studies [18].

The implementation of semiconductor gas sensor arrays as a sensing front-end for electronic noses resulted in multivariate data output that required complex pattern matching techniques for processing [19]. As a result, novel methods for signal pre-processing, dimensionality reduction, classification and regression were developed. Together, these formed the pattern recognition engine of an artificial olfactory system. Reviews [11,12] provided a detailed description of various methods and algorithms that were used in the pattern analysis for e-noses. The implementation of multi-stage pattern analysis methods required substantial computing power [20]. Hence, most of the e-nose systems adapting such pattern recognition engines required interfacing with powerful computers in order to run the pattern recognition algorithms. This resulted in limited portability of the e-nose device and hindered its application in a real-world environment.

Advancements in integrated-circuit technology and Micro-Electro-Mechanical Systems (MEMS) stimulated research into the development of portable electronic nose systems. The idea of a nose-on-a-chip discussed in [3] was first implemented in silicon by Tang and Goodman [21]. The initial design of the electronic nose chip mentioned in this paper comprised of an on-chip sensor unit, a signal processing unit, a database unit and a classifier unit. The sensing unit employed three carbon black-organic polymer sensors that output current signals in response to odour signatures. These represent odour concentration information and form a three-dimensional odour vector that is processed in further stages. Adaptive electronic circuits are implemented in the sensing stage to minimize baseline variations and other background noise. The signal processing unit operates in two states: the LEARNING state, where the normalized output vector is stored in an eight-bit static random access memory (SRAM) and the CLASSIFYING state, which includes the calculation of the Euclidean distance between the normalized signal vector and the data vector. The database unit is implemented using an SRAM to store and retrieve the normalized output signal vector during the LEARNING and CLASSIFYING states. The Euclidean distances between the signal and the data vector are compared and the resultant output is determined in the classifier unit. The chip was fabricated using the 1.2 µm 2-poly 2-metal process and was able to identify eight different odours.

Further study regarding the behaviour of the chip while interacting with analytes, its dependence on temperature variations and its power dissipation was published in [22]. A number of improvements were implemented, such as reducing the power dissipation by using analogue memory cells to store the information in analogue form to eliminate the power-hungry A/D and D/A converters. In the next stage of this research, Tang et al. [23] reported a portable electronic nose consisting of an array of eight commercial metal oxide sensors interfaced with an 8051-microprocessor board. The interface board operated in two modes: adaptation mode, in which the operating conditions are adjusted to a predefined baseline voltage; and sensing mode, in which analogue voltage output is translated to digital by A/D converters for further processing. The K-Nearest Neighbour (KNN) classification algorithm was implemented on the microprocessor and an accuracy in excess of 95% in identifying fruity odours was reported [23]. Inputs from these experiments were used to develop the next version of electronic nose system-on-chips (SoC). The e-nose chip reported in [24] was comprised of an integrated array of eight multi-walled nanotube (MWNT) sensors, an adaptive interface to cancel the baseline variations, a low-power 8-bit successive approximation register (SAR) ADC and a microcontroller implementing the KNN algorithm for classification. The e-nose chip, fabricated in a TSMC 0.18 µm 1P6M CMOS technology, consumed as little as 1.05 mW.

Along with advances in pattern-matching and processing methods such as multi-layer perceptron, Artificial Neural Networks (ANN), KNN, Principal Component Analysis (PCA) and cluster analysis [11,18,25], a considerable amount of research was directed towards improving the selectivity of sensor arrays by emulating the large number of receptor neurons in the biological pathway. Several studies implemented the temperature modulation technique to solve this issue [26,27,28]. This method is largely based on the principle of periodic sampling of sensor responses at varying temperatures. The temperature-dependent properties of the physical sensors were utilized to generate dimension-rich data at various temperatures, where each sensor is treated as a virtual sensor at all these different temperatures. The bio-inspired method for artificial olfaction proposed by Raman et al. in [26] utilized the temperature modulation technique for a 16-element metal oxide sensor array with a MEMS micro heater. The sensor array output was measured across a range of temperatures between 50 °C and 500 °C at a ramp rate of approximately 5 °C/s. A bio-inspired hierarchical processing method was used to sub-divide the classification tasks for odour categorization. Another application of temperature-modulation is mentioned in [27]. The authors implemented an oscillation-based temperature modulating technique where the sensor responses of a 16-element metal oxide sensor array were measured across a varying temperature range between 100 °C and 500 °C. The output is sampled every 0.2 s for a 150 s sinusoidal cycle to obtain 12,000 virtual sensors using 16 physical sensors.

Numerous other approaches have been reported that implemented a pattern-matching method for the identification and classification of odours [29,30,31,32,33,34,35]. Collectively, these studies indicate that the pattern-recognition engine forms an essential part of the existing electronic nose systems. Such a method usually consists of several computational stages where certain key parameters from the sensor information are extracted and utilized for the identification of the odours [12]. Substantial computing power is required to implement these methods, meaning they can only be implemented on computers, or microprocessors that can support the high computational requirements. Apart from being computationally expensive, the utilization of large computing devices restricts the portability of e-nose systems [36]. Although the e-nose systems implementing temperature modulation generated multi-dimensional data, the complexity involved in processing this large volume of data, coupled with the sampling methods, built up a considerable latency. This is among several factors that limited the application of most of the e-nose systems to a laboratory environment rather than real-world applications [10,37]. Other bio-inspired approaches such as fuzzy coding to determine odour concentration and identity information [38,39] were also reported.

## 3. Neuromorphic Olfactory Systems

The idea of neuromorphic engineering was proposed by Carver Mead in [40]. He highlighted the efficiency of neuro-biological systems while performing complex tasks such as motor movements based on visual/auditory sensing. This unconventional science takes inspiration from the computing principles of neuro-biological architecture to design analog Very-Large-Scale Integration (aVLSI) circuits. The attributes of neuromorphic systems such as spike-based sparse output generation and low-power consumption stimulated research to develop neuromorphic sensing systems that emulated the operating principles of biological sensory systems [37]. More recently, the implementation of neuromorphic engineering methods has contributed significantly towards the development of ultra-low power vision and auditory sensors such as the dynamic vision sensor (DVS) [41], the dynamic active vision sensor (DAVIS) [42] and the AEREAR [43].

The conventional e-nose systems were mainly data-driven, where the prime focus was to acquire detailed sensing information, select the odour-descriptive parameters and process this information for classification and identification [44]. However, neuromorphic methods focus on reducing the data by encoding only necessary information in the form of spikes, which simplifies the sensory information processing and allows implementation of learning algorithms.

The advantages offered by the neuromorphic approach coupled with recent progress in the understanding of the biological olfactory pathway stimulated application of these concepts for artificial olfactory systems [45]. In biology, a large number of Odour Receptor Neurons (ORNs)—that form the front-end of the mammalian olfactory pathway—are responsible for transduction of odour information in the form of spike trains. Information such as the membrane potential of the depolarized receptor neuron and the latency between spikes is encoded in the spike trains that enables the higher brain areas to process the information for identification and classification of odours [46,47].

The detailed review by Pearce in [18,48], explained the neuro-biological computations of the olfactory pathway and compared them with conventional pattern-matching methods to expose several avenues for future research in machine olfaction. These included the design of a hybrid sensing platform, emulating the dynamic range and wide selectivity of biological receptors, replicating the converging flow of sensory information in the biological olfactory pathway and implementing computational neuronal models instead of traditional pattern-matching. This further underpinned the need to apply novel bio-inspired methods for data representation and processing in machine olfaction.

### 3.1. Mammalian-Inspired Olfactory Systems

**Figure 2:**
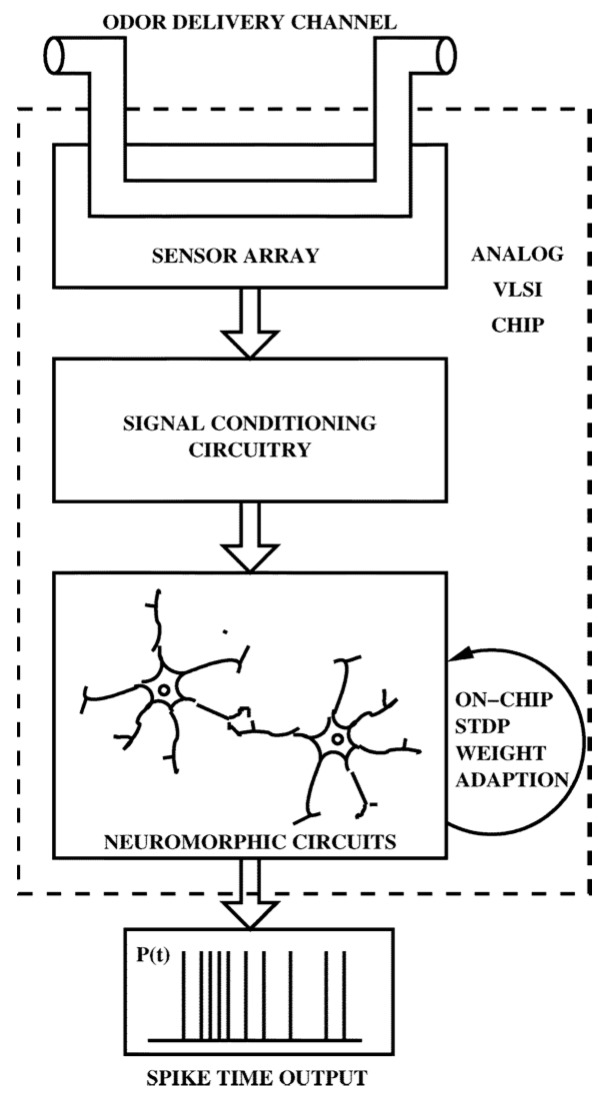
Block diagram of neuromorphic olfactory system proposed by Koickal et al. (Adapted from [16]).

Although progress towards implementing neuromorphic approaches for e-nose systems started in the early 2000s, the first such implementation in silicon was carried out by Koickal et al. in 2006 [49,50]. This aVLSI implementation comprised an on-chip chemosensor array, interface circuitry and a neuromorphic olfactory model integrated on a single chip fabricated using the AMS 0.6 μm CMOS technology (Figure 2) [16]. The heterogeneous chemosensor array integrated both chemFET and chemiresistive elements on a single platform [51]. Thin films of different carbon black (CB) polymers deposited across the 70 sensing elements of the array formed the chemically interactive material. However, the combination of chemFET and chemiresistive elements in a heterogeneous sensor array introduced undesirable baseline variations, mainly due to the requirements of different operating conditions and the poisoning effect during the post-processing of sensor elements [52]. Thus, a smart interface circuitry was designed and integrated with each sensor element to dynamically measure and cancel the variations in the baseline voltages.

The pre-processed chemosensory signals formed the input for the Olfactory Receptor Neuron (ORN) models that further translated these signals into spike-trains [53]. The projection neurons (PNs) form the second layer of the olfactory model. The spiking output from ORNs of similar types is integrated at a PN, which in turn drives the system output. The lateral inhibitory neurons implemented at this layer compete with other PNs to deliver improved selectivity through sharpened output patterns. One of the highlights of this olfactory model is the implementation of on-chip Spike-Time Dependent Plasticity (STDP) learning that improves the classification of odours using weight adaptations to learn odorant features [16].

While this research was effective in emulating major components of the biological olfactory architecture using neuromorphic methods, several issues – such as the component mismatch inherent in analog designs, compensation of baseline variation due to long-term drift and sensor poisoning and inconsistent behaviour of conducting polymer sensors under varying temperature and humidity conditions—were not addressed [54]. Although the shortcomings of this approach restricted the application of this model to solve real-world problems, it laid a strong foundation for future research in implementing neuromorphic models for artificial olfaction [49].

In contrast to the idea of emulating the entire biological olfactory pathway, research led by Bermak and colleagues [44,54,55,56,57,58,59] stressed the implementation of only the key computational principles that can be practically integrated in silicon. The CMOS chip designed by Ng et al. [54] utilized spike-latency encoding to develop novel solutions for gas identification in an e-nose system. A 4 × 4 tin-oxide gas sensor array, designed and fabricated using an in-house 5 μm, 2-metal, 1 poly process, formed the sensing front-end of a microelectronic nose. Each row of the sensor array, which consists of a metal catalyst (Pt, Ag, or Au), formed a “group” that exhibited similar drift behaviour. The ion implantation (B, P, or H) within each group created the sensor diversity required for broad selectivity and sensitivity to different target gases. A resistance-to-time conversion unit was implemented as part of the readout circuit that digitizes the resistance changes of each sensor element to spike trains. The methodology implemented for resistance-to-spike conversion cancelled the effect of concentration variations on the spike firing order and was proved by mathematical modelling in [55]. The spike pattern generated across each group of sensors corresponds to a unique 2-D rank order signature that can be defined for a specific gas. The processing unit compares the spatio-temporal spike pattern input to the pre-stored signatures in a reference library using simple XOR gates to identify the target gas.

This idea was further improved in [56] where the authors incorporated the biological concept of glomerular convergence by defining each group of sensors as a glomerulus. The latency between the spiking of each glomerulus is an elementary unit of the olfactory code [44,47]. Similar processing techniques using a reference library and a simple pattern-matching unit were applied to identify the target gases. The spike-latency coding for the identification of propane, ethanol and carbon dioxide achieved a corrected detection rate of 94.9%. This figure rose to 100% for glomerular latency coding [56,57]. Recent progress reported under this research includes the development of bio-inspired rank-order-based classifiers [58] and the implementation of weighted binary decision codes [60] for gas identification using a commercial gas sensor array.

The NEUROCHEM project, led by a number of European universities, started with the idea of modelling the vast selectivity and sensitivity of the olfactory epithelium of vertebrates and insects by developing a large-scale chemical sensor array [61]. The sensing front-end, composed of 65,536 sensor elements, was implemented using a modular approach in which each sensor module was comprised of 4096 polymer sensors. Broad and overlapping specificity to a wide range of volatile compounds was achieved by utilizing 31 different conducting polymer-based sensing materials. The read-out electronic circuit required two motherboards to drive and read the data from 16 sensor modules with a commercial National Instruments USB-6251board used for data acquisition and synchronization. The sensor data processing was based on software models of the vertebrate olfactory system that were developed and encoded in a custom GNU/Linux image. Although the biomimetic large-scale sensor array was a crucial step towards replicating the vast sensing capabilities of biological olfactory epithelium, the practical application of this system was limited, mainly due to the bulky design of the entire operational system restricting its portability [62].

### 3.2. Insect-Inspired Olfactory Systems

The olfactory path of insects has truly remarkable capabilities that enable them to perform highly specialized tasks such as finding mates, localizing food and detecting threats. More recently, researchers have taken great interest in replicating the neuro-computational architecture of the olfactory system of insects—especially the functionalities of antennal lobe (AL)—using aVLSI concepts [63]. Research by Beyeler et al. [64] explored the plausibility of a hardware emulation of the biological olfactory processing in the AL of the Drosophila melanogaster known generally as the common fruit fly. The proposed network architecture utilized the neurophysiological responses of the Drosophila’s ORNs extracted from an odorant response database as input data. The behaviour of the network was studied by implementing it using linear simulation, a software-based spiking simulation with the integrate-and-fire neurons and VLSI spiking emulation using two neuromorphic chips. This study demonstrated the importance of global feed-forward inhibition, showing that odour discriminability can be enhanced by increasing the vector-angles between odour pairs. As the prime focus of this research was the detailed bio-mimicry of the insect olfactory system and odour data transformations, the scope of application of this system for odour classification was very limited.

Research by Schmuker and Schneider [65] was directed towards the practical implementation of neurobiological computational principles of the insect olfactory system to develop neural networks for the processing and classification of odour data. The authors proposed a simplified three-stage processing and classifying architecture that mimicked the biological computation principles of the insect olfactory system. In the first stage, the behaviour of biological ORNs was implemented using “virtual receptors” that encode the stimulus odour data in the form of activation patterns. The correlation-based lateral inhibition among the glomerulus structures is implemented in the second stage to de-correlate the input vector. This further sharpens the responses of the virtual receptors to improve differentiation between similar odours. The third stage implements a pattern-matching technique—in this case a Naïve-Bayes classifier—and machine learning that assigns odour quality to the processed vector thereby aiding in the classification of odours.

Taking inputs from this research and the biological study of the honeybee’s olfactory system, Häusler et al. [66] developed a spiking neural network that replicated the multi-stage processing architecture of the honeybee’s olfactory pathway in order to resemble a deep-learning architecture. While the concepts applied for input data transformation and lateral inhibition are similar to those mentioned in [65], this research introduced supervised learning for the classification stage based on reward-dependent plasticity. This network was simulated using neuromorphic software tools such as PyNN and NEURON and the performance was comparable to a Naive Bayes classifier. Implementation of inhibitory Spike-Timing Dependent Plasticity (iSTDP) for unsupervised learning in the lateral inhibition was also explored in [67]. Later in the research, this network was deployed on Spikey [68], an aVLSI neuromorphic chip, to determine its performance in a real-world application. The authors highlighted the robust performance of the classifier network, despite variabilities such as temporal noise and device mismatch in the hardware system. As reported in [69], a classification accuracy of 87–96% was obtained for different sets of input data. More recently, further developments in this research utilized the responses of metal oxide sensors for training and input data. The spiking neural network, when simulated on a fast GPU and trained to identify 20 different chemical odours, delivered 92% accuracy within the first 30 s of exposure to the odour stimulus [70].

In summary, the neuromorphic trend in artificial olfactory systems started with the idea of emulating the working principles of the entire biological olfactory pathway. A number of studies [16,64,71,72] presented neuromorphic solutions for artificial olfaction with high levels of biorealism. The technical limitations and the complexity involved in mimicking the biological counterparts impeded the performance of these system for classification and identification of odours [54]. Due to these factors, research in neuromorphic olfaction either focused on modular developments (either sensing front-end [51,61,73,74] or processing unit [24,67,75,76,77,78]) or emulating only the neurobiological computation principle that can be practically implemented in silicon. The major contributions in neuromorphic olfaction are listed in Table 1. The application of neuromorphic concepts has enabled the encoding of large volumes of multivariate data into spike-based sparse information, thus considerably reducing the complexity involved in sensory data processing [79]. Moreover, the implementation of learning algorithms introduces robustness in neuromorphic olfactory systems against drift and other transient noise [16]. Current research in neuromorphic olfactory systems has a focus on applications to solve real-world problems in real-time with maximum accuracy.

## 4. Potential Sensing Front-Ends for Neuromorphic Olfaction

The sensing front-end is a crucial aspect of any e-nose system as it defines the signal conditioning and processing methods that can operate on the sensory response. As stated in [49], a neuromorphic sensing front-end that implements the neuronal functionality of ORNs is yet to be developed. Until recently, e-nose systems mostly employed either, or a combination of, metal oxide sensors and conducting polymer sensors, broadly classified as chemiresistive sensors, as sensing front-ends [5]. Recent advancements in material sciences and nanotechnology have led to the development of novel sensing technologies such as multi-walled carbon nanotubes (MWNT) [83] and gold nanoparticle [84]. As a result, recently developed sensing front-ends exhibit improved selectivity and sensitivity characteristics that can be used in conjunction with spike-based processing.

### 4.1. MEMS Sensors

A MEMS-based micro sensor array utilized for non-invasive detection of disease biomarkers through real-time breath monitoring is mentioned in [85]. The sensor array consists of 16 chemiresistive sensing elements arranged in a 4 × 4 configuration. Each sensor element is a micro-hotplate platform with different sensing material, mainly metal-oxides, deposited on them that introduces wide selectivity to different odorants. The ability to address each of the sensors individually is a key factor that can be exploited to integrate to an Address-Event-Representation (AER)-based interface while applying this sensing front-end in a neuromorphic olfactory system. A metal-oxide sensor, described in [86], based on similar micro-heater implementation can be utilized in an array configuration to generate activation patterns that can be deployed in a neuromorphic olfactory system.

### 4.2. MOX Sensors

Novel methods to improve selectivity, sensitivity and stability of traditional metal-oxide sensors have been actively researched. As the output response of metal-oxide sensors largely depends on the area exposed to the target gas, advanced nanofabrication methods are utilized to develop gas sensors with thin films of metal-oxide that enables a large area of the sensing film to be exposed to the target gas [87]. An e-nose system employing a thin film metal-oxide based gas micro sensor array is described in [88]. The front-end embedded system includes a customised A/D converter that can digitise minute resistance variations and a smart interface circuit to tackle drift and transient noise. The advantages of implementing a thin film metal-oxide gas sensor, such as quick response time and ability to extend selectivity through easy control of dopants, can be crucial in a neuromorphic olfactory system. The application of a spike-based approach and learning methods can further enhance the sensitivity and selectivity to substantially improve odour classification. Current research in the development of metal-oxide nanowire based sensors [89] has delivered promising results for potential application in micro-electronic noses in the near future.

### 4.3. CNT Sensors

**Figure 3:**
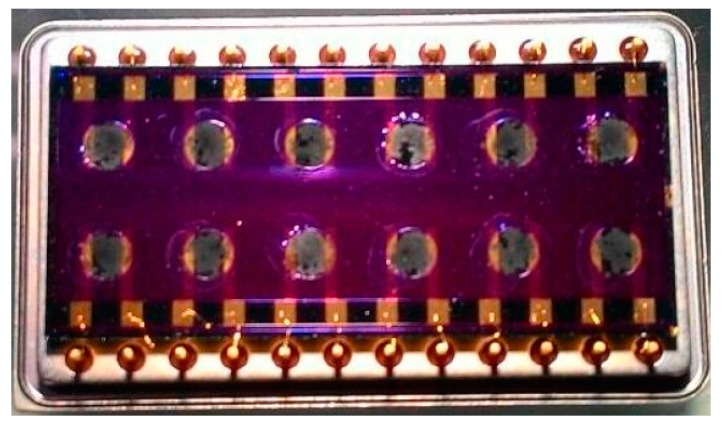
MWNT sensor array chip proposed by Wang et al. (Adapted from [92]).

Advancements in material science in conjunction with nanotechnology developments have resulted in increasing application of carbon nanotubes for electronic sensing, mainly due to its better conductivity properties than carbon black. The application of MWNT sensors in a portable electronic nose is described in [90,91,92]. The MWNT-polymer-sensor array chip (Figure 3) comprised of eight independent sections with coatings of different polymer sensing materials. The undesirable effects of temperature and humidity variations and background odours were minimized by implementing a bio-inspired fast readout circuit that was interfaced with the sensor array chip.

A previously tested traditional pattern-matching algorithm [23] was implemented for odour identification. There is promising scope to improve these outcomes through integration with a neuromorphic processor, such a neuromorphic olfactory system can vastly benefit from the salient features of the CNT-polymer composite sensors such as ultra-sensitivity, quick response time, reproducibility and long-term stable output. This research group also explored the properties of single-walled carbon nanotube networks and assessed its performance for a gas sensing application in [93]. Several other studies have investigated the application of CNTs for gas sensing implementations [83].

### 4.4. Front-End and Pre-Processing Integration

Traditionally, key functionalities of gas sensing front-ends were limited to A/D conversion and signal conditioning [94]. A recent study towards development of a bio-inspired analogue front-end is reported in [73]. The authors proposed an integrated bio-inspired parameter extraction technique that encodes key gas-identifying parameters into spike patterns. The implementation of online learning methods enables adaptation to varying conditions that aids in determining shift in some parameters due to transient noise. The 6-channel analogue front-end, with a metal-oxide gas sensor array, is fabricated using TSMC 65 nm low-power CMOS technology. The power consumption of the front-end is as low as 463 nW/channel in the normal mode and 519 nW/channel in the training mode. The application of such a front-end in a neuromorphic olfactory system can substantially reduce the overhead of applying parameter extraction and dimensionality reduction methods as a part of the processing unit. Furthermore, the spike-based output of the front-end can contribute towards reducing latency due to signal transformation during the processing stage.

## 5. Conclusions and Future Research

E-nose technology has evolved drastically in the last decade, mainly due to the advancement of computing methods complemented by developments in material sciences. The application of bio-inspired computing techniques in e-nose devices brought a complete paradigm shift from the traditional single-channel gas sensing methods. The introduction of sensor arrays enhanced the selectivity and sensitivity of the sensing front-end, thus generating dimension-rich multi-variate data that could be processed using bio-inspired pattern-matching techniques [26]. Recent advancements in neuromorphic engineering led to the development of a new generation of e-noses that attempts to emulate the biological olfactory pathway.

The application of neuromorphic approaches to artificial olfactory systems has two advantages. Firstly, the representation of large volumes of multivariate data in spikes enables efficient encoding of critical information such as time-to-spike and odour concentration. This further reduces the processing overhead by avoiding the dimensionality reduction and feature extraction stages [56]. Secondly, the spike-based representation of sensory data enables implementation of learning algorithms that aids in minimizing undesired effects of thermal noise and drift [77].

Initially, neuromorphic olfactory systems mainly focused on emulating all the aspects of the biological olfactory pathway at a high level of abstraction. However, these systems failed to demonstrate substantial improvement in performance for classification and identification of odours [56]. Further research in neuromorphic olfaction focused on emulating only the underlying biological computational principles that can be integrated in a chip. This resulted in several modular developments where the prime focus was to either develop a sensing front-end with spike-based output or a spike-based processing unit. Over the last decade, numerous developments in neuromorphic olfactory systems have been reported but unfortunately only a few of these implementations have demonstrated feasibility for a real-world application [79]. While certain limitations of neuromorphic olfactory systems, such as improving signal-to-noise ratio and minimizing the effect of long term drift, are being actively researched, the real-time operation of these systems has not really been addressed in current studies [59]. The exposure time required by most of the neuromorphic olfactory systems for reliable odour identification causes considerable latency [79]. Implementing learning methods and parallel processing across multiple input channels [95] are a few of the possible solutions that can enable the olfactory system to determine at least the class of the odour in real-time. For critical applications such as bio-security, even the identification of odour class is of paramount importance.

The equivalent research in neuromorphic vision and auditory sensors has been underpinned by the performance benchmarks set by the key contributions such as DVS and AEREAR [37]. In most cases, the performance of the neuromorphic olfactory systems has been determined under ideal laboratory conditions [54,96,97]. The accuracy of such systems when exposed to real-world data with considerable background noise cannot be evaluated for benchmarking. The implementation of a robust neuromorphic olfactory system by integration of modular developments can contribute towards determining performance benchmarks for olfactory systems.

Biological research has demonstrated the correlation between olfaction and gustation and the existence of computational parallels in higher brain areas [98]. The advancements in neuromorphic olfactory processing have exposed an interesting avenue for research where similar computational principles can be applied to develop neuromorphic gustatory sensors. Traditionally, electronic tongues have been mainly applied to determine tastes of different compounds [99]. With appropriate sensing front-ends, the application of spike-based neuromorphic gustatory sensors can be extended to determine the chemical composition of liquids. More recently, implementation of e-noses in conjunction with e-tongues have been reported for applications such as food quality assessment [99,100,101,102]. Based on similar concepts, correlation of neuromorphic gustation and olfaction can expose numerous research avenues for future work.

## Figures and Tables

**Table 1:** Major contributions in neuromorphic olfaction.

Published Date	Authors	Contribution	Reference
July 2006	Raman et al.	Biologically inspired olfactory coding model	[80]
January 2007	Koickal et al.	aVLSI adaptive neuromorphic olfaction chip	[16]
May 2007	Guerrero-Rivera and Pearce	Olfactory bulb model using spiking FPGA	[72]
October 2007	Schmuker and Schneider	Three-layered processing and classification model of insect olfactory system	[65]
November 2010	Beyeler et al.	Software simulation and hardware model of the AL of the Drosophila melanogaster	[64]
May 2011	Hausler et al.	Deep learning SNN based on the olfactory system of the honeybee	[66]
July 2011	Ng et al.	A CMOS gas recognition chip based on 2D spatio-temporal spike signatures	[54]
January 2012	Yamani et al.	Extension of [54] by implementing glomerular latency coding	[56]
June 2012	Imam et al.	Emulation of mammalian olfactory glomerular layer using digital neuromorphic chip	[71]
July 2012	Hsieh and Tang	SNN chip based on subthreshold oscillation and onset latency for odour classification	[77]
November 2012	Bernabei et al.	Large-scale biomimetic sensor array (NEUROCHEM project)	[74]
July 2013	Pearce et al.	Neuromorphic spiking model of the insect antennal lobe macro glomerular complex	[81]
November 2013	Kasap and Schmuker	Unsupervised learning based on iSTDP in a SNN inspired by the insect AL	[67]
December 2013	Schmuker et al.	Implementation of a SNN described in [65] on *Spikey*, a neuromorphic hardware system	[69]
January 2016	Diamond et al.	Implementation of bioinspired SNN described in [69] for classification of real-time sensor data	[70]
November 2016	Jing et al.	Bioinspired signal processing method for e-noses based on olfactory bulb model	[82]
